# Manipulation of Pro-Sociality and Rule-Following with Non-invasive Brain Stimulation

**DOI:** 10.1038/s41598-018-19997-5

**Published:** 2018-01-29

**Authors:** Jörg Gross, Franziska Emmerling, Alexander Vostroknutov, Alexander T. Sack

**Affiliations:** 1 Institute of Psychology, Leiden University, Leiden, The Netherlands; 2 Center for Experimental Economics and Political Decision Making, University of Amsterdam, Amsterdam, The Netherlands; 3 Department of Cognitive Neuroscience, Maastricht University, Maastricht, The Netherlands; 4 Department of Experimental Psychology, University of Oxford, Oxford, United Kingdom; 5 Center for Mind & Brain Sciences, University of Trento, Trento, Italy

## Abstract

Decisions are often governed by rules on adequate social behaviour. Recent research suggests that the right lateral prefrontal cortex (rLPFC) is involved in the implementation of internal fairness rules (norms), by controlling the impulse to act selfishly. A drawback of these studies is that the assumed norms and impulses have to be deduced from behaviour and that norm-following and pro-sociality are indistinguishable. Here, we directly confronted participants with a rule that demanded to make advantageous or disadvantageous monetary allocations for themselves or another person. To disentangle its functional role in rule-following and pro-sociality, we divergently manipulated the rLPFC by applying cathodal or anodal transcranial direct current stimulation (tDCS). Cathodal tDCS increased participants’ rule-following, even of rules that demanded to lose money or hurt another person financially. In contrast, anodal tDCS led participants to specifically violate more often those rules that were at odds with what participants chose freely. Brain stimulation over the rLPFC thus did not simply increase or decrease selfishness. Instead, by disentangling rule-following and pro-sociality, our results point to a broader role of the rLPFC in integrating the costs and benefits of rules in order to align decisions with internal goals, ultimately enabling to flexibly adapt social behaviour.

## Introduction

Rules play a vital role in human societies. Adhering to speeding limits, not littering, or customs like shaking hands help to organise and regulate everyday life. Rules often demand to restrict goal-directed behaviour. For example, waiting in front of a red traffic light or standing in a queue in the supermarket interferes with the internal goal to proceed towards one’s destination, or to not waste more time than strictly necessary.

Likewise, in the social domain, norms about fairness, morality, or pro-sociality often demand to restrict selfishness. The right lateral prefrontal cortex (LPFC) has been causally linked to the implementation of pro-social norms^1–7^. For example, brain stimulation, both with transcranial magnetic stimulation (TMS)^3,8^ and transcranial direct current stimulation (tDCS)^9^ over the right LPFC led to higher acceptance rates of unfair offers in the Ultimatum Game. In this game, participants have to make the decision to accept or reject an offer from another participant about splitting a sum of money. In case of rejection, both participants earn nothing. Applying cathodal stimulation, believed to decrease excitability of neurons in the targeted brain region^10,11^, increased the propensity to accept highly unequal and thus unfair offers.

One interpretation of these findings, that has been put forward, is that participants under cathodal TMS and tDCS were less able to resist the economic temptation to accept low offers, since ‘something is still better than nothing’^1–7,9,12–14^. Resonating with this interpretation, participants under cathodal TMS also made faster decisions^14^, which was interpreted as a sign for a quick uncontrolled selfish impulse guiding decision-making. At the same time, anodal tDCS over the right LPFC, believed to increase excitability of neurons in the targeted brain region^10,11^, led to more social norm compliance^13^. From this perspective, the right LPFC exhibits executive control over the impulse to act selfishly and allows to align behaviour with social norms and rules.

A different functional role of the right LPFC in normative social decision making has been recently put forward by Buckholtz^15^. Rather than simply implementing ‘impulse control’, it is argued that the LPFC is involved in a value based cost-benefit analysis by weighing and integrating the costs and benefits of actions, rules, personal goals, past experience, and other situational factors like frames. In line with this interpretation, the LPFC has been broadly associated with adaptive behaviour that enables humans to flexibly react to external stimuli in order to implement internal goals, rather than just follow fixed stimulus-response patterns or arbitrary rules^12,16–21^ and integrates thought and action in the pursuit of these goals^18–25^.

Resonating with this interpretation, while brain stimulation over the right LPFC shifted decisions towards more selfishness or more pro-sociality depending on the stimulation, it did not affect the underlying fairness perception^9,12–14^. This suggests that brain stimulation over the right LPFC led to a misalignment of thought and action. Further, Greene *et al*.^26^ have shown that lying (i.e. breaking a norm) exhibits LPFC activity, while honesty did not and FeldmanHall *et al*.^27^ found a positive correlation between LPFC activity and the extent of selfishness. Both findings are at odds with the selfish impulse control hypothesis of LPFC recruitment. Also, difficult moral dilemmas, that require to find a compromise between norms and welfare maximisation, have been associated with greater LPFC activity^28^, pointing to a value-based integrative function of the LPFC.

Here, we aim to experimentally disentangle the role of the LPFC in following rules that either restrict payoff maximisation (similar to fairness norms) or not, using transcranial direct current stimulation (tDCS). In the experiment, participants repeatedly choose to maximise their own or the payoff of another person. In one part of the experiment, participants are free to choose, and we can hence observe their unrestricted behaviour as a proxy for internal motives or goals. In another part, we confront participants with a rule that demands which option to choose. The rule is sometimes aligned with what participants would have chosen without a rule (i.e. the rule coincides with their unrestricted behaviour or internal goals), is consequence neutral, or demands to choose an action that does not coincide with their unrestricted behaviour.

If the right LPFC is critically involved in suppressing selfish impulses, decreasing neural excitability of this brain area with cathodal stimulation should lead to more rule violations when the rule demands to restrict payoff maximisation, while anodal stimulation should lead to more rule-following, even when the rule demands to restrict payoff maximisation. Whereas if the right LPFC plays a broader role in aligning behaviour with internal goals, anodal brain stimulation should lead to more rule violations when the rule is at odds with what participants would choose without the rule (i.e. the rule is in conflict with internal goals), while decreasing neural excitability with cathodal stimulation should lead to more ‘blind’ rule-following, due to the impeded ability to integrate internal goals and actions consequences in the decision.

We further let participants make decisions in series of mini dictator games in which participants have to distribute a sum of money either selfishly or pro-socially. Again, we measure unrestricted behaviour under all three tDCS condition and compare it to a situation when a rule is in place that demands to either take the pro-social or selfish option.

## Methods

### Subjects

Participants were recruited from the subject pool of the Behavioral and Experimental Economics Lab (BEElab) at Maastricht University and were invited via e-mail. Experiments were conducted with the informed consent of 103 healthy adult subjects (mean age = 21.4 +/−3.0, 56 female) who were free to withdraw from participation at any time. The study was approved by the local ethical committee of the Faculty of Psychology and Neuroscience, Maastricht University and all methods were performed in accordance with the relevant guidelines and regulations.

### Experimental Procedure

**Figure 1:**
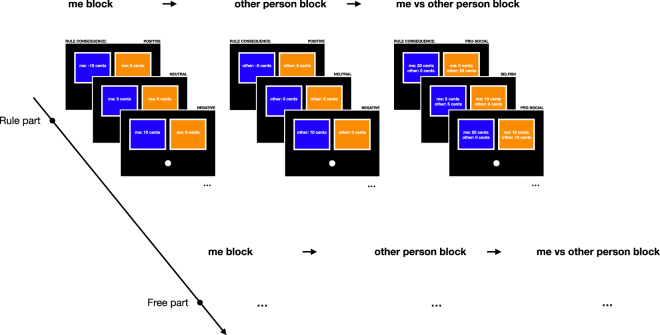
Experimental design. Participants repeatedly had to drag a ball to either the blue or orange box. Whether the decision had real financial consequences for either the participant (‘me’ block), another person (‘other person’ block), or both (‘me vs. other person’ block) changed across rounds. In the ‘free’ part, participants freely decided to drag the ball in either box, whereas in the ‘rule’ part a simple rule was given to the participant. In the ‘rule’ part, half of the participants were instructed to always place the ball in the blue box, whereas the other half was instructed to always place it in the orange box. The rule would be ‘always place the ball in above the orange box’ in the illustrated case had positive, negative or neutral consequences, or demanded to take the selfish or pro-social option.

Upon arriving at the lab, participants were seated in individual cubicles in front of a computer screen. Four to six participants completed the experiment simultaneously to ensure that they trusted their decisions to impact another real human individual. In the experiment, participants had to decide repeatedly whether to drag a ball with the mouse to either the left or right side of the computer screen into a blue or orange box (Fig. 1).

Across three blocks, the decisions had real financial consequences either for the participant (‘me’ block), for another real but unknown person (‘other person’ block), or both, the participant and another person (‘me vs. other person’ block). For example, in a given trial of the ‘me’ or ‘other person’ block, dragging the ball to the blue box would yield 10 cents, while dragging the ball to the orange box would yield 0 cents for the participant or the other person, respectively.

In each trial of the ‘me vs. other person’ block participants had two options to distribute a sum of money between themselves and the other person (so called mini dictator game). For example, dragging the ball to the blue box would yield 10 cents for the participant but 0 cents for the other person, while dragging the ball to the orange box would yield 5 cents to the participant and 5 cents for the other person.

In one part of the experiment, participants could freely choose to opt for the action they preferred (‘free’ part). In the other part, a simple and arbitrary rule was given to the participants (‘rule’ part). The rule was to always drag the ball either to the blue or orange box (counterbalanced across participants), regardless of the consequence (“The rule is to put each ball in the blue (orange) area”). The order of blocks (‘me’ block, ‘other person’ block, ‘me vs. other person’ block) and parts (‘free’ part, ‘rule’ part) were fully crossed and the order counterbalanced across participants.

After finishing the main task, participants made a series of fairness judgements in which a hypothetical person A distributed a sum of money between herself and another hypothetical person B. Participants rated each allocation on a fairness scale from −3 (completely unfair) to 3 (completely fair). After answering demographics questions, participants were finished. Participation took around 40 minutes. At the end of the experiment, the sum of money was paid to both the participant and the other person, according to the decisions the participant made in the experiment.

### Decision consequences

**Figure 2:**
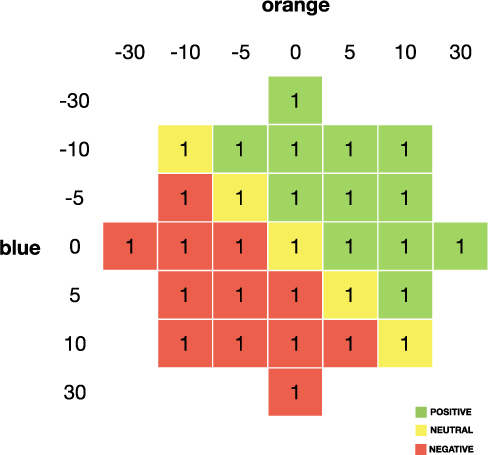
Payoff consequences. All possible combinations of outcomes in the ‘me’ block and ‘other person’ block. Assuming that the rule was to put each ball in the orange box in the ‘rule’ block, the rule sometimes demanded to choose the positive option (green), a neutral option (yellow), or a negative option (red).

Dragging the ball to either the blue or orange box could lead to the following consequences in euro-cents: [−30, −10, −5, 0, 5, 10, 30] in the ‘me’ block and ‘other person’ block. Figure 2 shows all trial combinations each participant was confronted with in each block and part.

When a rule was in place (‘rule’ part), the rule demanded to choose the *positive* option in 12 trials (e.g. choosing 10 cents over 5 cents), to choose a *neutral* option in 5 trials (e.g. choosing 5 cents over 5 cents), or to choose a *negative* option in 12 trials (for example choosing 5 cents over 10 cents) for oneself or the other person in the ‘me’ block or ‘other person’ block, respectively (see Fig. 2).

In each trial of the ‘me vs. other person’ block participants had two options to distribute a sum of money between themselves and the other person (Fig. 1). The sum of money could take the following values in euro-cents: [−30, −20, −10, 10, 20, 30], and could be distributed in the following way:

−30: [−30,0], [0,−30],

−20: [−20,0], [−10,−10], [0,−20],

−10: [−10,0], [−5,−5], [0,−10],

10: [10,0], [5,5], [0,10],

20: [20,0], [10,10], [0,20],

30: [30,0], [0,30]

Participants had to make choices for all possible combinations of these allocations (in total 28 trials in both, the ‘free’ part and the ‘rule’ part). Participants, thus, repeatedly faced the option to either distribute a sum of money more selfishly (taking a bigger share of the money), or more pro-socially (giving more or splitting the amount equally). When a rule was in place (‘rule’ part), the rule demanded to choose the selfish option in half of the trials (e.g. 10 cents for oneself and 0 cents for the other person). In the other half of the trials, the rule demanded to choose the pro-social option (e.g. 5 cents for oneself and 5 cents for the other person). Trial order was randomised across participants.

### Correlates of rule-following

The rule-following task as used in the ‘me’ block has been shown to predict normative behaviour in other experimental games and correlates with political orientation^29^. In particular high rule-followers exhibit higher and sustained cooperation in social dilemmas^30^, more reciprocity of trust^30^, more pro-social behaviour in dictator games^31^, and more honesty in a cheating game (unpublished data). Importantly, the behaviour of rule-followers in these tasks is correlated with normative judgements about what “one should do”^30^. Further, rule-following decreases with externally administered Oxytocin, possibly due to biasing reward processing vs. norm-following tendencies, through the mesolimbic pathway^32^.

The rule-following task implemented in this study differs from previous studies in two points: (a) The monetary consequence of violating vs. following the rule changes from trial to trial and (b) in some parts of the experiment, the decisions have consequences for another person, or both another person and the decision maker. Previous studies have shown that about one third of participants follow the rule unconditionally^29,30,32^, meaning that they place the ball in the box according to the rule across all trials. Arguably, unconditional rule-followers simply follow a fixed behavioural pattern without perceiving a conflict between what they want to choose and what the situation dictates them to do. Previous studies have shown that brain stimulation over the LPFC has little effect on behaviour in this case^12,33^. We, hence, expect little behavioural changes in unconditional rule-followers under tDCS and decided a priori to focus our main analysis on participants who react to the different incentives of the choice options across trials (conditional rule-followers).

### tDCS manipulation

**Figure 3:**
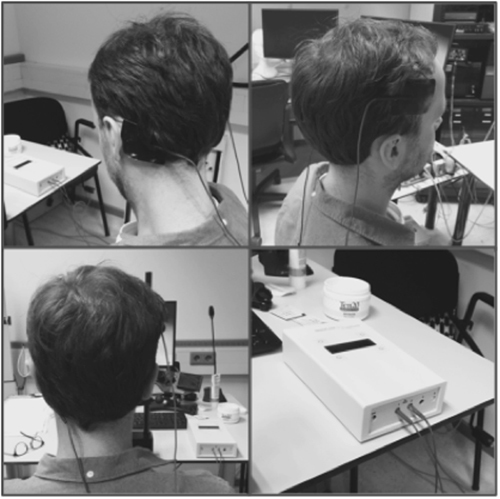
tDCS setup. Anode or cathode placed over F4 (international 10/20 EEG system), reference over contralateral mastoid.

To test the involvement of the right LPFC on rule adherence, we used a double-blind placebo-controlled tDCS design. Participants (n = 103) were randomly assigned to three tDCS conditions. TDCS is a non-invasive brain modulation technique using micro-currents believed to manipulate the resting membrane potential of neurons in the targeted brain region^34–36^. In a placebo/sham condition (n = 36) the skin sensations accompanying real stimulation can be mimicked, while no real stimulation is administered. Therefore, participants cannot differentiate the sham condition from real modulation. The right LPFC was manipulated with either cathodal (n = 32) or anodal tDCS (n = 35) over F4 as determined by the international 10/20-EEG system (Fig. 3; electrode sites were localised using an easy cap system). The reference electrode was placed over the respective contralateral mastoid. TDCS (neuroConn, Ilmenau, Germany) was applied by 5 × 7 cm standard electrodes, at an intensity of 2 mA, and with 30 s ramping phases. Stimulation was applied to all participants participating in a session, during the entirety of the task execution (30 minutes). No manipulation was induced in the sham condition. There was no significant difference in in the distribution of sex (chi square test, *χ*^2^(3) = 0.37, p = 0.83) or age (one-way ANOVA, F(2) = 15.7, p = 0.43) across tDCS condition.

## Results

### Unrestricted behaviour

**Figure 4:**
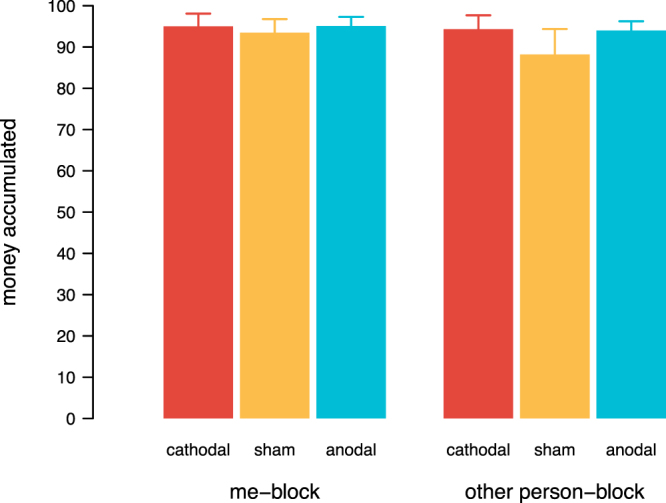
Free decisions. Money accumulated for oneself (‘me’ block) or another person (‘other person’ block). 100% is the maximum that can be earned by always choosing the option that would yield more. Error bars show the standard errors of the mean.

To analyse whether brain stimulation affected internal goals or intrinsic behaviour, we first looked at the decisions of participants when they were free to choose and not confronted with a conflict between social motives (selfishness vs. pro-sociality) – i.e. the ‘me’ block and the ‘other person’ block under no rule. In these blocks, participants simply had to decide whether to maximise their own (‘me’ block) or another person’s payoff (‘other person’ block) by choosing the better option. We calculated the sum of money each subject accumulated across these two blocks when no rule was present and entered the data into a censored regression model fitted in R. Unsurprisingly, during sham, participants overwhelmingly chose options that would yield the most money for themselves or the other person. This was not significantly altered by the two active stimulation conditions, showing that participants were still able to identify and willing to choose the option that is most beneficial for themselves or the other person during cathodal and anodal tDCS (Fig. 4, Tables S1 and S2). Hence, tDCS did not significantly alter participants’ free choices across these two blocks.

### Rule-Following

**Figure 5:**
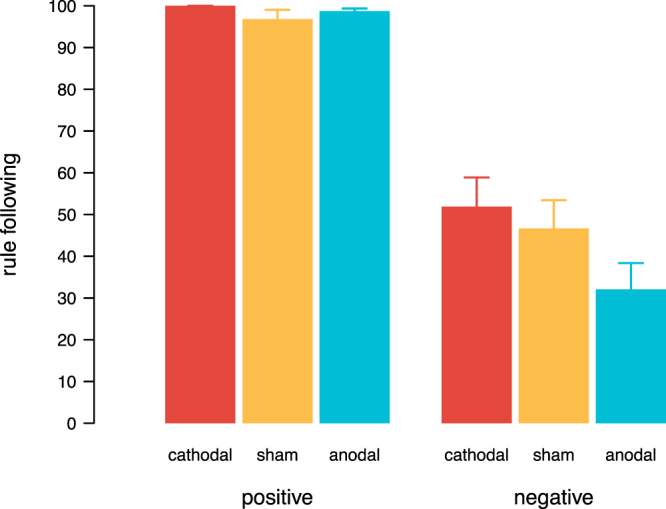
Rule-following. Average rule-following in percentage when following the rule had positive consequences, i.e. demanding to take the option that would yield more money to oneself (‘me’ block) or another person (‘other person’ block), or when the rule had negative consequences, i.e. demanding to take the option that would hurt oneself or the other person financially. Error bars show the standard errors of the mean.

To analyse whether brain stimulation affected rule adherence, in particular when the rule demanded to restrict intrinsic payoff maximising behaviour, we looked at the decisions of participants when they were confronted with a rule in the ‘me’ block and ‘other person’ block. When a rule was in place, 36 participants followed the rule unconditionally independent of the tDCS condition (chi square test, *χ*^2^(2) = 1.46, p = 0.48). We, hence, focused on those subjects, who violated the rule depending on the decision consequences and reacted to the incentives of the task (for a model incorporating also unconditional rule-followers, see Supplementary Material). Across the two blocks, conditional rule-followers followed the rule nearly without exception when it had positive consequences, and hence did not conflict with intrinsic behaviour (Fig. 5). When adhering to the rule led to negative consequences and, thus, did not coincide with what participants chose freely, rule adherence dropped from 98% to 44%, averaging across all brain stimulation conditions (Wilcoxon Signed Rank test, W = 6198, p < 0.001). However, participants under cathodal tDCS still followed the rule 52% of the time, while it was only followed 32% of the time under anodal tDCS (Fig. 5, Mann–Whitney U test, U = 630, p = 0.02). Decisions under cathodal and sham tDCS did not differ significantly (Mann–Whitney U test, U = 1021, p = 0.55).

**Figure 6:**
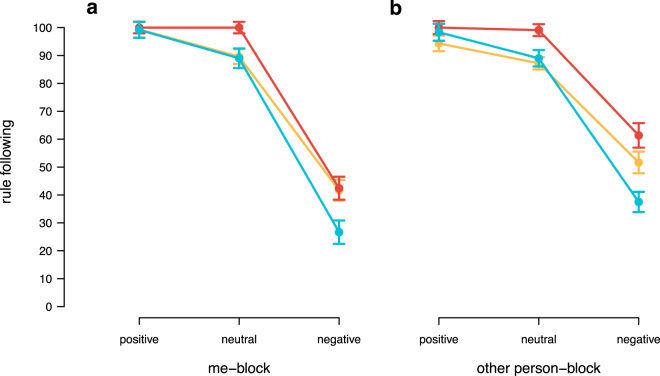
Rule-following across blocks depending on the consequence. Average rule-following in percentage across tDCS conditions (red = cathodal tDCS, yellow = sham, blue = anodal tDCS) when the consequence of the rule was either negative (following the rule led to a worse outcome), neutral (following or violating the rule led to the same outcome), or positive (following the rule led to a better outcome), separately for decisions that either affected the participant herself (**a**) or another person (**b**). Error bars show the standard errors of the mean.

This pattern was consistent across ‘me’-trials (Fig. 6a), and ‘other person’-trials (Fig. 6b). We aggregated the number of times participants followed the rule for the ‘me’ block and the ‘other person’ block for each type of consequence: negative (meaning that the rule demanded to take the negative option), neutral (meaning that following the rule yielded the same outcome as violating the rule), and positive (meaning that the rule was to take the option that would benefit oneself or the other person financially). Thus, we had three values for each participant in each block, measuring the average obedience to the rule. To account for the dependencies within subjects, we fitted two (Bayesian) random intercept regression models using JAGS/R to the ‘me’-trial and ‘other person’-trial data, respectively. Non-informative Gaussian priors (m = 0, sd = 100) were used for each predictor and non-informative uniform priors (range 0 to 100) for the error terms. We used three parallel chains. For every estimated coefficient, the potential scale reduction factor (Gelman and Rubin Diagnostic) was below 1.05, indicating good mixing of the three chains and, thus, high convergence. Regression tables reported below show estimated coefficients together with the 95% confidence interval (CI). Fitting the models using restricted maximum likelihood (REML) as implemented in the lme4 package in R revealed similar estimates and the same statistical inferences.

While participants overwhelmingly followed the rule when it was beneficial to them or the other person across all three tDCS conditions, participants under cathodal compared to anodal tDCS followed more of those rules that were in conflict with intrinsic behaviour, both when the participant herself had to bear the consequences (random-effects regression, reduction in rule-following for negative consequences, 95% CI: [−0.31,−0.01], Table 1) or when another person was affected (random-effects regression, reduction in rule-following for negative consequences, 95% CI: [−0.40,−0.08], Table 2), with sham being in the middle, but not significantly different from the stimulation conditions.

**Table 1:** ‘Me’-trials (confronted with a rule).

	Estimate	SD	95% CI
intercept (cathodal tDCS – negative)	0.42	0.053	[0.32, 0.523]
sham tDCS	−0.01	0.073	[−0.15, 0.13]
anodal tDCS	−0.16	0.077	[−0.31, −0.01]
neutral consequence	0.58	0.071	[0.44, 0.71]
positive consequence	0.58	0.071	[0.43, 0.71]
sham tDCS × neutral	−0.10	0.097	[−0.29, 0.09]
anodal tDCS × neutral	0.05	0.103	[−0.15, 0.25]
sham tDCS × positive	0.01	0.097	[−0.19, 0.19]
anodal tDCS × positive	0.15	0.103	[−0.06, 0.35]
random intercept variance	0.08	0.034	[0.00, 0.13]

Random intercept regression predicting the propensity to follow rules in ‘me’-trials, depending on the tDCS condition.

**Table 2:** ‘Other person’-trials (confronted with a rule).

	Estimate	SD	95% CI
intercept (cathodal tDCS – negative)	0.61	0.057	[0.51, 0.73]
sham tDCS	−0.10	0.078	[−0.25, 0.06]
anodal tDCS	−0.24	0.083	[−0.40, −0.08]
neutral consequence	0.38	0.069	[0.24, 0.51]
positive consequence	0.39	0.070	[0.25, 0.52]
sham tDCS × neutral	−0.02	0.094	[−0.21, 0.16]
anodal tDCS × neutral	0.14	0.100	[−0.06, 0.33]
sham tDCS × positive	0.04	0.095	[−0.15, 0.22]
anodal tDCS × positive	0.22	0.100	[0.03, 0.42]
random intercept variance	0.13	0.027	[0.08, 0.19]

Random intercept regression predicting the propensity to follow rules in ‘other person’-trials, depending on the tDCS condition.

Importantly, if cathodal tDCS of the right LPFC lowers the control of ‘selfish impulses’, we should have seen more rule violations when the rule was in conflict with maximising personal payoff in the ‘me’ block under cathodal vs. anodal tDCS. Instead, we observe the opposite; more rule-adherence under cathodal tDCS.

### Selfishness changes due to rule-following

So far, we focused our attention on rule-adherence when the decision only involved maximising or sacrificing money for oneself (‘me’ block) or another person (‘other person’ block). To more directly test the effect of rules that demand to restrict selfishness, we looked at rule-adherence when the decision further involved a trade-off between maximising money for oneself *at the expense* of another person (‘me vs. other person’ block).

For each participant, we calculated selfishness as a function of accumulated money. In each ‘me vs. other person’ block, participants could accumulate up to 180 euro cents for themselves, by always choosing the best option for themselves. Because they played zero-sum dictator games, the other person would lose 180 euro cents in this case. We define this choice pattern as 100% selfishness. By choosing to always distribute gains and losses equally and fairly across oneself and the other person, both participants would earn 0 euro cents. We define this choice pattern as 0% selfishness, i.e. maximum equality.

**Figure 7:**
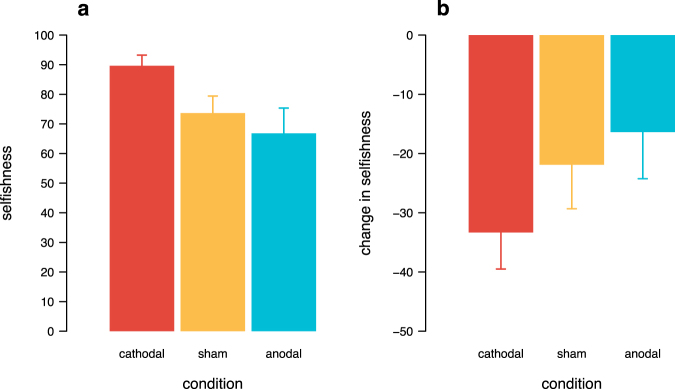
Selfishness and change in selfishness due to following the rule. (**a**) Average amount of money accumulated for oneself at the expense of another person across tDCS conditions in the ‘free’ part (0% corresponds to an equal and fair split of the money, 100% means maximal selfishness). (**b**) Change in selfishness, as measured by the difference in accumulated earnings between the ‘free’ part and the ‘rule’ part, when faced with a rule that demanded to take the pro-social option in half of the trials. Error bars show the standard errors of the mean.

When choosing freely, participants accumulated significantly more money for themselves under cathodal tDCS of the right LPFC compared to anodal tDCS of this brain area (Fig. 7a, Mann-Whitney U test, U = 404, p = 0.04). Thus, we replicated the previously observed effect that cathodal tDCS over the right LPFC leads to more selfish decisions^3,6,9,12–14^.

To analyse how being faced with a rule changed selfishness, we looked at the change in earnings that participants accumulated at the expense of the other person when a rule was in place. Note that the rule demanded to choose the selfish option in half of the trials and the pro-social option in the other half of the trials (i.e. demanded 0% selfishness from the participant as defined above). Selfishness was significantly reduced under cathodal compared to anodal tDCS (Fig. 7b, Mann–Whitney U test, U = 307, p = 0.03). The rule, that dictated more pro-social decisions as compared to what participants chose freely in the ‘free’ part (Fig. 7a), led participants to give 33% more to the other person on average (and thus took 33% less for themselves), during cathodal stimulation of the right LPFC. Thus, the confrontation with a rather pro-social rule was able to attenuate the increased selfishness of participants under cathodal tDCS, while participants stayed more consistent with their free choices under anodal tDCS (Fig. 7b).

### Fairness evaluations

**Figure 8:**
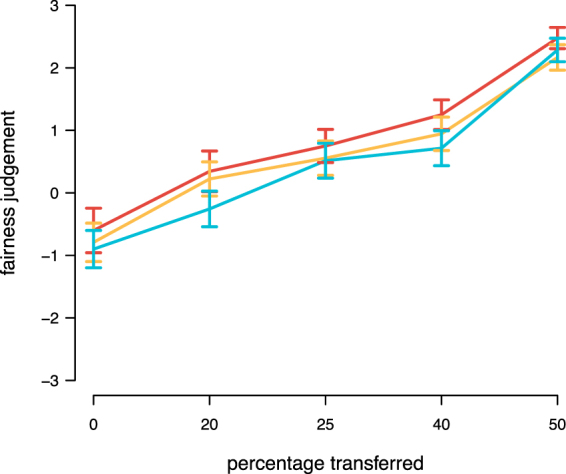
Fairness judgements. Average fairness judgements on a scale from −3 to 3 depending on the amount transferred by a hypothetical person A to a hypothetical person B for each tDCS treatment (red = cathodal tDCS, yellow = sham, blue = anodal tDCS).

After the main task, participants made fairness judgements for several hypothetical money allocations between a person A and a person B. Neither cathodal, nor anodal tDCS altered the fairness perception of participants (Fig. 8 and Table S3). In line with earlier findings^12–14,37^, this suggests that brain stimulation led participants to make different decisions without changing the underlying evaluation process.

## Discussion

Rules often take the form of external restrictions on the pursuit of own goals, and sometimes demand to take actions that are against one’s own will. We often follow rules, nevertheless^38^. Here we provided evidence for a causal involvement of the right LPFC in rule-following with social consequences. When freely deciding, participants made decisions that would yield the most benefits for them or others and manipulating the right LPFC did not change that. When an arbitrarily imposed rule coincided with this internal goal, people followed the rule overwhelmingly, irrespective of brain stimulation. However, when the rule was to hurt oneself or another person financially, anodal tDCS over this brain area led to more rule breaking, compared to more rule-following under cathodal tDCS.

At the same time, under cathodal tDCS, participants made rather selfish choices in allocating a sum of money between them and another person. Being confronted with a rule that demanded to split the money more pro-socially, selfishness was, however, significantly reduced. Thus, although cathodal tDCS led to more damage towards oneself or another person due to high rule-following of a costly rule, a rather ‘pro-social’ rule in the ‘me vs. other person’ block was able to make participants choose the socially desirable option more often. Under anodal tDCS on the other hand, participants stayed more consistent with their free choices when a rule was in place. In sum, independent of whether the rule demanded to exhibit more pro-social behaviour, to restrict payoff maximisation, or to hurt another person financially, decisions under cathodal tDCS were more guided by rule-following, while decisions under anodal tDCS were more aligned with what participants would have chosen without a rule, based on incentives and decision consequences.

Following the hypothesis in the literature, that cathodal stimulation to the right LPFC leads to more selfish payoff maximisation^3,9,12–14^, we should have seen more rule-breaking under cathodal tDCS and increased selfishness, regardless of the rule. Instead our results suggest that the right LPFC is critically involved in shifting behaviour from a more rule-based decision mode that is less sensitive to consequences (i.e. hurting another person or being pro-social towards another person) to decisions that are more focused on outcomes and consequences in light of internal goals. This result is in line with the idea that the LPFC is important for a cost-benefit integration of external restrictions and own goals, rather than controlling selfish impulses^15^.

By disentangling the role of rules in pro-social decisions with regards to the LPFC, and demonstrating that tDCS can systematically modify the alignment between internal goals and external restrictions, these results may be able to reconcile seemingly contradictory observations and opposing views in the literature regarding the automaticity of pro-social behaviour. While selfishness has been seen as the impulse that needs to be controlled by executive control instances^8,9^, on the flipside, some scholars argued that pro-social behaviour is impulsive and rational-economic reasoning towards payoff maximisation is controlled by secondary control processes^40–42^. Further, some studies have observed lower pro-social behaviour after cathodal brain stimulation^6,8,9,12^, while others have observed higher pro-social behaviour^2,5,13^.

Our results also resonate with a recent brain stimulation study showing that anodal tDCS over the right LPFC increases honesty when honesty is in conflict with material gain^33^. Based on our results and interpretation, this finding may be explained by internal goals (honesty) that are in conflict with the economic temptation to cheat. This conflict is resolved in favour of internal goals (honesty), when anodal tDCS is applied. As in previous studies, the internal goals or intrinsic behaviour have to be deduced post-hoc and we hence can only speculate about it, while in our design we can directly compare behaviour under no rule and rule when the rule is either aligned or in conflict with intrinsic behaviour. Maréchal *et al*.^33^ further found no difference of tDCS in participants that cheated to the extreme. This resonates with our finding that unconditional rule-following is not affected by tDCS, and suggests that individual differences exists in the extent to which a situation is perceived as a conflict between motives that needs trading off (and therefore the recruitment of the right LPFC) or not. While we find differences between the two active brain stimulation protocols, the difference to the sham condition were smaller and not significant. Further, while we demonstrate a causal effect of the two active tDCS conditions over the right LPFC on rule-following with social consequences, we do not know how the manipulation of the resting membrane potential in the LPFC interacts with other brain areas like the vMPFC, ACC, or subcortical reward areas that have been implicated in value-based decision making^43–47^. Future studies may be needed to investigate this further.

Both, phylogenetically and ontogenetically, the LPFC is one of the latest developing brain regions^48–51^ and its major role has been implied in adaptive behaviour that enables humans, as compared to other vertebrates, to flexibly react to external stimuli in order to implement goals, rather than just follow fixed stimulus-response patterns^12,16–21^. Following rules regardless of its consequence can be seen as just reacting to an external stimulus, whereas weighing the costs and benefits of a rule, and deciding to follow it depending on its consequences, is arguably a much more adaptive behaviour. We demonstrate that the right LPFC is involved in trading off internal goals with external restrictions, helping us to violate rules when they just demand to hurt someone without any other benefits. These results may further our understanding of the neurobiological basis of normative decision making in the social domain. Instead of a simple dichotomy of subcortical brain areas that drive selfishness, and the LPFC controlling these ‘selfish impulses’, our results provide a more nuanced explanation of the function of the LPFC in human social behaviour, in line with the broader cognitive literature, suggesting that the right LPFC plays a crucial part in flexibly reacting to the social environment by trading off internal goals with external restrictions.

## Electronic supplementary material

Supplementary Information
