# Supplementary material for: Manipulation of Pro-Sociality and Rule-Following with Non-invasive Brain Stimulation
Source: Sci Rep. 2018 Jan 29;8:1827. doi: 10.1038/s41598-018-19997-5 (PMC5789006; doi:10.1038/s41598-018-19997-5)
Supplement: Supplementary file 1 — Supplementary Information [file 41598_2018_19997_MOESM1_ESM.pdf]

Supplementary Information

Manipulation of Pro-Sociality and Rule-Following with Non-invasive Brain Stimulation

Jörg Gross, Franziska Emmerling, Alexander Vostroknutov, Alexander Sack

Experimental Interface

In each trial, participants had to drag a ball in the middle of the computer screen to, either, a blue or yellow box. The consequence of this decision changed across trials and blocks. Figure S1 shows two example screenshots of the computer interface for the 'me' block / 'other person' block (Figure S1a) and the 'me vs. other person' block (Figure S1b).

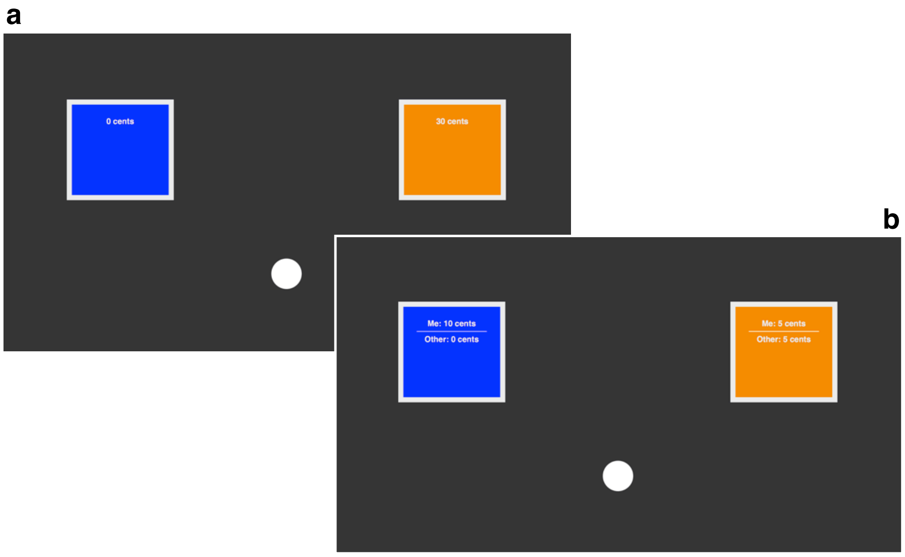

Figure S1. Interface screenshots. Participants repeatedly had to drag a ball to either the blue or orange box. In the 'me' block and 'other person' block, the decision had real financial consequences for the participant or another person, respectively, that changed across trials (see **a** for an example-trial). In the 'me vs. other person' block, participants had to decide between allocating a sum of money between themselves and another person. The sum, as well as the allocation-choice changed across rounds (see **b** for an example-trial).

Free Decisions in the 'me' and 'other person' block

Table S1 and S2 show the regression results, estimating the accumulated money for oneself ('me' block), or another person ('other person' block) across the three tDCS conditions.

Table S1.
'Me'-trials (free decisions).
Censored regression predicting the earnings for oneself in the 'free' part, depending on the tDCS condition.

	Estimate	Std. error	t-value	p-value
Intercept (cathodal tDCS)	242.56	28.18	8.61	< 0.01
sham tDCS	-14.04	28.96	-0.49	0.63
anodal tDCS	-23.62	28.66	-0.82	0.41

Table S2.
'Other person'-trials (free decisions).
Censored regression predicting the earnings for the other person in the 'free' part, depending on the tDCS condition.

	Estimate	Std. error	t-value	p-value
Intercept (cathodal tDCS)	238.81	28.70	8.32	< 0.01
sham tDCS	-16.90	31.13	-0.54	0.59
anodal tDCS	-6.71	31.45	-0.21	0.83

Fairness Judgements

Table S3 shows the regression results for the fairness evaluations depending on tDCS condition, the hypothetical transfer, and its interaction.

Table S3.
Fairness evaluations.
Random intercept regression predicting fairness ratings depending on different money allocations, and tDCS condition.

	Estimate	SD	95% CI
Intercept (cathodal tDCS)	-0.73	0.19	[-1.09, -0.36]
sham tDCS	-0.20	0.26	[-0.72, 0.30]
anodal tDCS	-0.39	0.26	[-0.90, 0.11]
percentage transferred	6.04	0.34	[5.39, 6.72]
sham tDCS x percentage transferred	-0.20	0.47	[-1.10, 0.72]
anodal tDCS x percentage transferred	0.15	0.47	[-0.80, 1.05]
random intercept variance	0.88	0.08	[0.74, 1.04]

Additional Analysis

We specifically wanted to test the role of the right LPFC in rule-following when the rule did not coincide with what participants would choose in the 'free' part (i.e. rules that demanded to financially hurt oneself or the other person), while showing that behaviour is unchanged when internal goals and the rule coincide (i.e. rules that are beneficial or neutral).

However, due to aggregating the data across consequences we lost possibly valuable variability related to the degree of how beneficial or harmful following the rule really was (see experimental setup & design).

We therefore also fitted two more complex models to the non-aggregated data, using the binary trial-by-trial response variable (0 = not following the rule, 1 = following the rule). As predictor, we used the continuous rule consequence variable, that varied between -30 (following the rule would lead to a loss of 30 cents) and +30 (following the rule would lead to earning 30 cents more than violation the rule). In this regression, we included the observations of all participants and dummy-coded unconditional rule-following (participants who followed the rule across all trials without being influenced by its consequence at all).

To account for the dependencies within subjects, we fitted two (Bayesian) random intercept binomial regression models using JAGS/R to the 'me'-trial and 'other person'-trial data, respectively. Non-informative Gaussian priors (m = 0, sd = 100) were used for each predictor and non-informative uniform priors (range 0 to 100) for the error terms. We used three parallel chains. For every estimated coefficient, the potential scale reduction factor (Gelman and Rubin Diagnostic) was below 1.05, indicating good mixing of the three chains and thus high convergence. Regression tables reported below show estimated coefficients (log-odds) together with the 95% confidence interval (CI, also called highest density interval in the Bayesian framework). Note that, since non-informative priors were used, a 95% CI that only contains negative or positive values can be interpreted as significant at a p = .05 two-sided threshold from a frequentist perspective. Fitting the models using restricted maximum likelihood (REML) as implemented in the lme4 package in R revealed similar estimates and resulted in the same statistical inferences.

Table S4 and Figure S2a show the fitted model for 'me'-trials. As can be seen, the probability to follow the rule increased the more beneficial the rule was, up to 100% for rules that would yield beneficial outcomes to the participant (consequence ≥ 0) in all three tDCS conditions. However, participants under cathodal and sham tDCS, compared to anodal tDCS, had a higher likelihood to follow harmful rules and, therefore, had a steeper increase in rule obedience towards more beneficial consequences.

Table S5 and Figure S2b shows the fitted model for 'other person'-trials. Again, the probability to follow the rule increased the more beneficial the rule was, up to 100% for rules that would yield beneficial outcomes to the other person (consequence ≥ 0) in all three tDCS conditions. However, participants under cathodal, compared to anodal, tDCS had again a higher likelihood to follow harmful rules.

Table S4.
'Me'-trials ('confronted with a rule).
Random intercept binomial regression predicting the likelihood to follow rules in 'me'-trials, depending on the tDCS condition.

	Estimate	SD	95% CI
Intercept (cathodal tDCS)	3.36	0.72	[1.94, 4.78]
sham tDCS	-0.21	0.97	[-2.13, 1.68]
anodal tDCS	-0.66	1.02	[-2.65, 1.34]
lost by following	0.25	0.02	[0.20, 0.29]
full adherence	31.02	16.78	[11.96, 68.89]
sham tDCS x lost by following	-0.01	0.03	[-0.07, 0.05]
anodal tDCS x lost by following	0.07	0.03	[0.01, 0.14]
random intercept variance	3.06	0.38	[2.37, 3.85]

Table S5.
'Other person'-trials (confronted with a rule).
Random intercept binomial regression predicting the likelihood to follow rules in 'other person'-trials, depending on the tDCS condition.

	Estimate	SD	95% CI
Intercept (cathodal tDCS)	5.23	0.94	[3.46, 7.15]
sham tDCS	-2.28	1.19	[-4.63, 0.04]
anodal tDCS	-2.59	1.23	[-5.14, -0.26]
lost by following	0.23	0.03	[0.18, 0.28]
full adherence	29.82	17.05	[9.82, 67.62]
sham tDCS x lost by following	-0.07	0.03	[-0.12, -0.01]
anodal tDCS x lost by following	-0.01	0.03	[-0.07, 0.05]
random intercept variance	3.46	0.47	[2.66, 4.48]

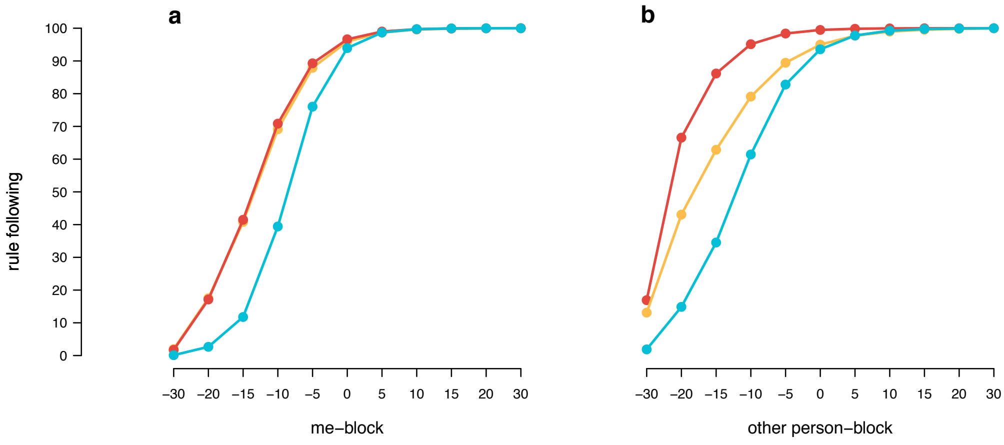

Figure S2. Predicted probability to follow the rule as a function of the consequence of rule-following (red = cathodal tDCS, yellow = sham, blue = anodal tDCS), when the consequences of the rules would affect the participant (**a**) or another person (**b**).

Selfish vs. pro-social rules

In the main manuscript, we focus on selfishness (money accumulated for oneself at the expense of another person) when participants were free to decide across tDCS conditions and looked at how selfishness was attenuated by a rule that dictated to take the pro-social choice in half of the trials.

To further disentangle rule-following in the 'me vs. other person' block, we separately looked at rule-following when the rule was selfish (e.g. the rule dictated to take 10 cents and give 0 cents to the other person instead of taking 5 cents and giving 5 cents) vs. when the rule was pro-social (e.g. the rule dictated to take 5 cents and give 5 cents to the other person instead of taking 10 cents and giving 0 cents). We compared this to the intrinsic behaviour of participants in the 'free' part (i.e. percentage of selfish vs. pro-social choices).

Figure S3 separately shows average selfishness when freely deciding and selfishness when the rule dictated to be selfish (a), and average pro-sociality when freely deciding and pro-sociality when the rule dictated to be pro-social (b), across tDCS condition.

When freely deciding, participants under cathodal tDCS chose the selfish option more frequently (64.7%) compared to participants under anodal tDCS (54.4%), with sham tDCS in the middle (57.7%). All participants frequently followed the rule, when it dictated to take the selfish option, especially under cathodal tDCS. Participants under cathodal tDCS followed a 'selfish rule' 98.3% of the time, followed by sham tDCS with 91.7% and anodal tDCS with 89.3% (see Figure S3a). Interestingly, across all conditions, participants increased their

selfish choices when the rule dictated them to be selfish, indicating that a selfish rule can serve as an excuse to act selfishly.

When the rule was to take the pro-social option, participants under cathodal tDCS changed their intrinsic behaviour the most, as can be seen by the difference between voluntary pro-social choices vs. rule-induced pro-social choices (Figure S3b). On the contrary, participants under anodal tDCS chose the pro-social option in 45.5% of the trials on average when freely deciding and only marginally deviated from their voluntary behaviour when a rule dictated them to choose the pro-social option (44.6%).

Taken together, this pattern led to the highest attenuation of selfishness under cathodal tDCS. While participants accumulated more money for themselves at the expense of the other person under cathodal vs. anodal tDCS when freely deciding, participants under cathodal tDCS accumulated significantly less money when confronted with a rule that dictated pro-social choices in 50% of the trials, while participants under anodal tDCS stayed more consistent with their free choices (Figure S4).

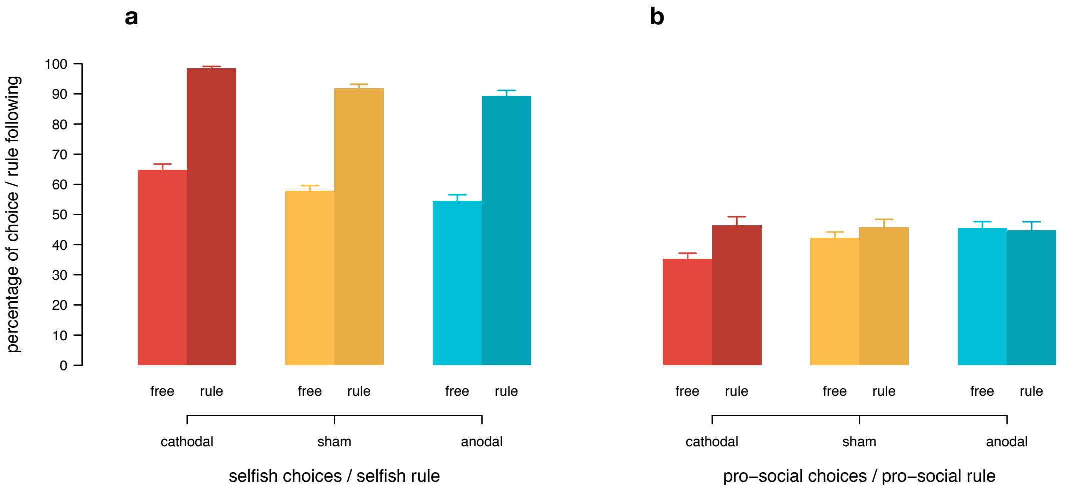

Figure S3. Average percentage of free selfish choices (light bars) and percentage of selfish choices when the rule dictated to be selfish (**a**) and average percentage of free pro-social choices (light bars) and percentage of pro-social choices when the rule dictated to be pro-social (**b**), across tDCS condition (red = cathodal tDCS, yellow = sham tDCS, blue = anodal tDCS).

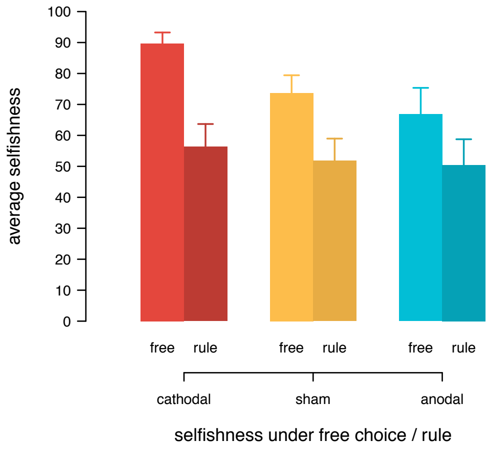

Figure S4. Average selfishness (percentage of maximum possible earnings for oneself) when freely deciding (light bars) and when a rule dictated behaviour, across tDCS condition (red = cathodal tDCS, yellow = sham tDCS, blue = anodal tDCS).
